# Bilateral visual impairment caused by *Toxoplasma gondii* encephalitis and ocular GVHD in a patient after allo-HSCT

**DOI:** 10.1186/s12348-026-00582-1

**Published:** 2026-04-28

**Authors:** Armin Taghavi Eraghi, Uwe Pleyer, Tina Dietrich-Ntoukas

**Affiliations:** Department of Ophthalmology, Charité – Universitätsmedizin Berlin, Berlin, Germany

## Abstract

**Background:**

Cerebral toxoplasmosis is a rare but potentially fatal opportunistic infection in non-HIV immunocompromised patients, particularly after allogeneic hematopoietic stem cell transplantation (allo-HSCT). Diagnosis is often delayed due to nonspecific clinical manifestations.

**Case presentation:**

We report a 66-year-old man with myelodysplastic syndrome that had transformed into secondary acute myeloid leukemia. Following remission induction with CPX-351, he underwent allo-HSCT and received ciclosporin A, mycophenolate mofetil, and corticosteroids for graft-versus-host disease prophylaxis. Standard cotrimoxazole prophylaxis was withheld because of hepatic intolerance and thrombocytopenia, and inhaled pentamidine was administered instead. Three months post-transplant, the patient presented with bilateral visual field deficits. Automated perimetry revealed a homonymous inferior quadrantanopia. Brain MRI demonstrated a solitary ring-enhancing lesion in the left optic radiation, and cerebrospinal fluid PCR confirmed *Toxoplasma gondii*. Treatment with sulfadiazine, pyrimethamine, and folinic acid was initiated, followed by long-term secondary prophylaxis. Despite residual visual impairment, the patient achieved clinical stability.

**Conclusion:**

This case underscores the diagnostic value of neuro-ophthalmologic examination and early neuroimaging in detecting opportunistic CNS infections. It also highlights the challenges of prophylactic decision-making in immunocompromised patients when cotrimoxazole is contraindicated. Individualized immunosuppressive and prophylactic strategies, along with multidisciplinary care, were key to successful management.

## Introduction

Cerebral toxoplasmosis is an uncommon but often fatal opportunistic infection in recipients of allogeneic hematopoietic stem cell transplantation (allo-HSCT), typically caused by reactivation of latent *Toxoplasma gondii* during profound post-transplant T-cell dysfunction and intensified immunosuppression for graft-versus-host disease [1–4]. Reported post-allo-HSCT incidences vary according to population seroprevalence and local practices, with single-centre series reporting rates of approximately ≈ 4% and a characteristic early onset within the first three months after transplant [2, 3]. From an ophthalmic standpoint, toxoplasmosis may present with retinochoroiditis or neuro-ophthalmic deficits resulting from lesions along the visual pathway; visual field loss can be an early clue that prompts neuroimaging and directed testing [1, 5–7]. Diagnosis is challenging because clinical and radiologic features overlap with fungal abscess, nocardiosis, or post-transplant lymphoproliferative disorder; detection of T. gondii DNA by PCR in cerebrospinal fluid or blood allows rapid confirmation [1, 4]. Contemporary guidance recommends trimethoprim–sulfamethoxazole (TMP-SMX) prophylaxis for seropositive recipients; however, myelotoxicity may limit early use, and alternative Pneumocystis regimens (e.g., aerosolized pentamidine) lack anti-Toxoplasma activity— necessitating either prophylaxis with active alternatives or pre-emptive PCR monitoring during periods of severe lymphopenia [1, 4, 5, 7, 8]. Heightened vigilance among ophthalmology, hemato-oncological and infectious diseases teams is therefore critical for timely recognition, targeted therapy, and prevention in this high-risk population [1–4].

## Case

A 66-year-old man with a history of myelodysplastic syndrome (MDS) with isolated 5q deletion, initially treated with lenalidomide, progressed to secondary acute myeloid leukemia (sAML) im April 2021. He achieved complete remission following induction and consolidation with CPX-351 and was referred for allogeneic hematopoietic stem cell transplantation (allo-HSCT). During chemotherapy, he developed sepsis due to multidrug-resistant E. coli and vancomycin-resistant Enterococcus, along with transient candidemia.

In August 2021, he underwent allo-HSCT from a matched unrelated donor after conditioning with treosulfan, fludarabine, and antithymocyte globulin (ATG). Graft-versus-host disease (GvHD) prophylaxis included ciclosporin and mycophenolate mofetil. Early post-transplant complications included 3MRGN E. coli sepsis (day + 7), oral mucositis, and mild hyperbilirubinemia. Engraftment was achieved by day + 15. Around day + 18, he developed Grade II acute cutaneous GvHD, initially responsive to topical steroids but later requiring systemic corticosteroids.

At discharge on day + 29, the patient was clinically stable, with partial GvHD response. Laboratory tests showed pancytopenia, elevated CRP, and mildly impaired renal function. Anti-infective prophylaxis included acyclovir, letermovir, and posaconazole. Cotrimoxazole was withheld due to thrombocytopenia, and inhaled pentamidine was administered for Pneumocystis prophylaxis. Immunosuppressive therapy was continued, and outpatient monitoring focused on immune reconstitution.

**Fig. 1:**
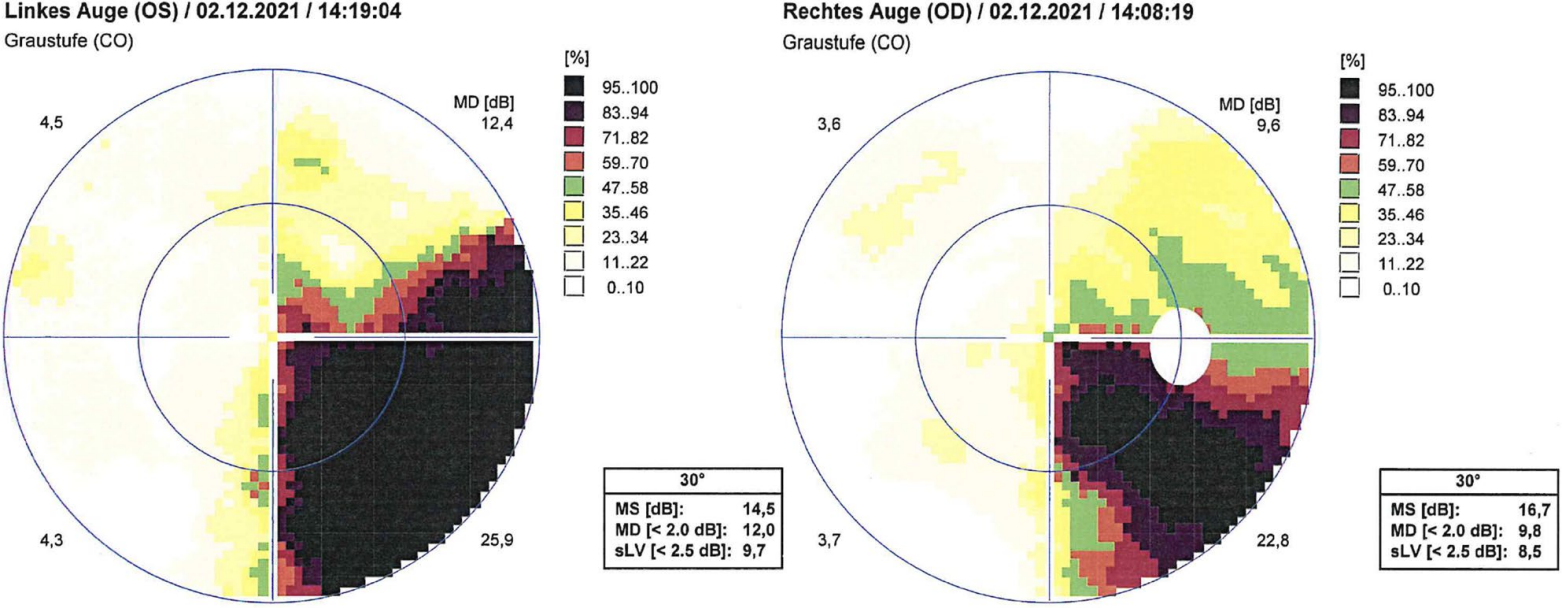
Humphrey 30 − 2 visual field testing demonstrating a right homonymous inferior quadrantanopia with bilateral inferonasal and inferotemporal field loss (mean deviation 12.0 dB [OS] / 9.8 dB [OD])

Three months post-transplant, during routine follow-up for ocular GVHD screening, the patient reported visual disturbances. Ophthalmological evaluation revealed a homonymous inferior quadrantanopia (Fig. 1) characterized by bilateral inferonasal and inferotemporal field loss (MD -12.8 dB / -11.8 dB), and reduced visual acuity (OD 0.16, OS 0.3). Intraocular pressure and pupillary responses were normal. Slit-lamp examination showed minor corneal changes but no clear evidence of ocular GvHD yet. Fundoscopy was unremarkable. No signs of posterior segment inflammation (vitritis or retinochoroiditis) were observed, and serial fundus examinations during subsequent follow-up remained unremarkable. Based on these findings, a central neurological lesion was suspected, and the patient was readmitted to the hematology–oncology ward for further evaluation.

**Fig. 2:**
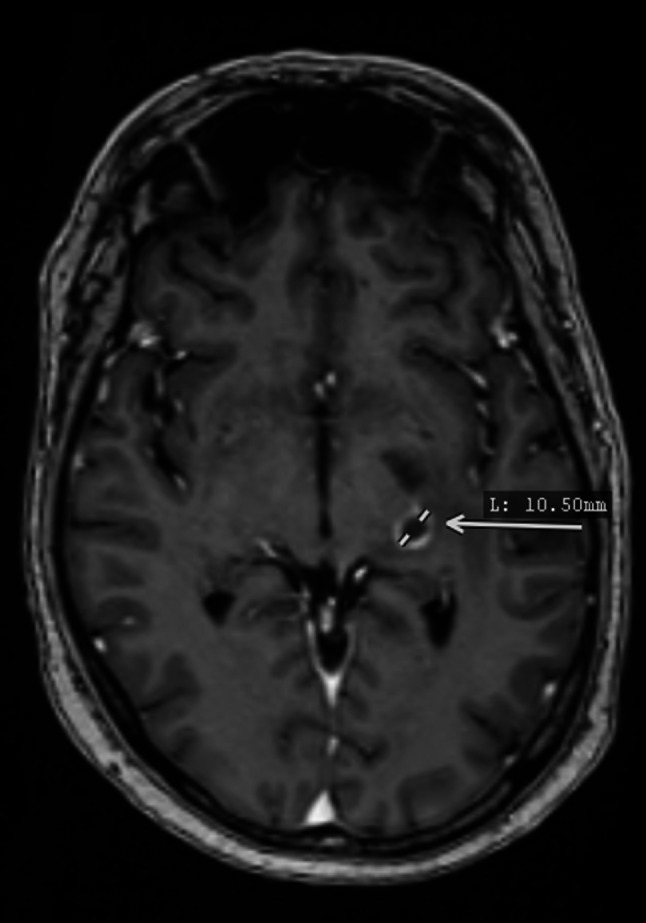
Axial contrast-enhanced T1-weighted MRI demonstrating a 10.5 mm ring-enhancing lesion in the left optic radiation, associated with mild vasogenic edema and corresponding to the patient’s visual field defect

Brain MRI (Fig. 2) performed the same day demonstrated a 10 mm ring-enhancing lesion in the left optic radiation with vasogenic edema, corresponding to the visual field defect.

Neurological consultation later that day confirmed the quadrantanopia and revealed additional symptoms of fatigue, confusion, and a bilateral postural–intention tremor. At that time, he was receiving ciclosporin A and methylprednisolone, but no cotrimoxazole due to persistent thrombocytopenia. Anti-infective prophylaxis included acyclovir, letermovir, and posaconazole.

Laboratory tests showed elevated CRP (35.1 mg/L), ferritin (1903 µg/L), and gamma-GT (230 U/L), along with leukopenia (1.82/nL) and CD4⁺ lymphopenia (126/µL), indicating severe immunosuppression. CMV and EBV PCRs were negative.

Differential diagnoses included cerebral toxoplasmosis, cryptococcosis, and post-transplant lymphoproliferative disorder (PTLD). Empirical therapy was initiated with pyrimethamine (200 mg loading dose, then 75 mg daily), sulfadiazine (1.5 g QID), and folinic acid (15 mg daily), together with liposomal amphotericin B (240 mg/day) and flucytosine (2 g QID).

Lumbar puncture revealed elevated CSF protein (681 mg/L), mild pleocytosis (6 cells/µL), and increased lactate (27 mg/dL). CSF PCR was positive for *Toxoplasma gondii*, confirming cerebral toxoplasmosis; other pathogens were excluded. Targeted therapy continued with sulfadiazine (1.5 g Q6h or 3 g Q12h), pyrimethamine (75 mg daily), and folinic acid (25 mg daily). Supratherapeutic sulfadiazine levels necessitated dose reduction to 4 g/day with close monitoring.

In February 2022 (six months post-HSCT), he was re-admitted with fatigue, anorexia, nausea, and weight loss. He was cachectic (ECOG 3, Karnofsky 60%) and fully dependent. Labs showed hypokalemia (K⁺ 2.2 mmol/L), hypocalcemia, hypoalbuminemia (21.2 g/L), hypogammaglobulinemia (IgG 3.8 g/L), and CD4⁺ lymphopenia (126/µL). GI-GvHD was suspected but ruled out on endoscopy and histology. Parenteral nutrition was initiated.

A follow-up MRI on February 16 demonstrated regression of the cerebral lesion. Therapy was de-escalated to secondary prophylaxis with sulfadiazine (2–3 g/day), pyrimethamine (25–50 mg/day), and folinic acid, planned through one-year post-transplant or immune reconstitution. During this admission, asymptomatic CMV reactivation (12,200 copies/mL) was treated with ganciclovir. Transient hyponatremia (Na⁺ 129 mmol/L) was managed conservatively. A brief episode of sterile cystitis resolved spontaneously.

Neuro-ophthalmic follow-up confirmed persistence of the right homonymous inferior quadrantanopia without clinical progression. Repeat automated perimetry after treatment was not available in the clinical record.

At discharge on March 30, 2022, the patient was clinically stable, receiving nutritional support, and remained in complete hematologic remission with full donor chimerism.

In March 2025, the patient returned with bilateral visual decline and ocular surface discomfort. Ophthalmic examination revealed signs of chronic ocular GvHD (punctate keratopathy, corneal scarring, corneal ulceration), meibomian gland dysfunction, and reduced tear production [9]. Visual acuity had declined to OD 0.08 and OS 0.16.

He was treated with topical corticosteroids, ciclosporin A (Cequa), ofloxacin, and intensive lubrication. A bandage contact lens was applied to the left eye because of deep corneal ulceration. Oral doxycycline was initiated for its anti-inflammatory effects (reduction of matrix metalloproteinase activity in the corneal stroma). This regimen led to gradual epithelial healing and improved visual acuity (OD 0.16, OS 0.32). Despite ocular stability by May 2025, the patient reported new‑onset vertigo and cognitive symptoms.

Re-evaluation raised concern for relapse of cerebral toxoplasmosis or PTLD. He had discontinued anti-toxoplasma therapy three months earlier (February 2025) at his own request. Although no longer receiving immunosuppressive therapy, his CD4⁺ count remained severely depressed (0.4/nL). Neurological examination was unchanged, but Montreal Cognitive Assessment (MoCA) testing revealed mild cognitive slowing (24/30).

MRI demonstrated mild enhancement in the left optic radiation. Cerebrospinal fluid (CSF) analysis showed mild pleocytosis, elevated protein, and negative PCR results for Toxoplasma, JC virus, EBV, and HIV. No malignant cells were present. Tau and β-amyloid levels were borderline, without evidence of neurodegeneration. These findings were attributed to post-infectious leukoencephalopathy. Multidisciplinary outpatient surveillance was continued.

## Discussion

This case underscores the diagnostic value of neuro-ophthalmologic examination and early neuroimaging in detecting opportunistic CNS infections. Post-chiasmal visual pathway involvement may be the presenting manifestation of cerebral toxoplasmosis after allo-HSCT. The patient developed a right homonymous inferior quadrantanopia and reduced visual acuity three months after transplantation; MRI revealed a ring-enhancing occipital lesion with surrounding edema, and CSF PCR confirmed *Toxoplasma gondii*. Early antiparasitic therapy resulted in radiological regression, while the visual field defect remained clinically stable; repeat automated perimetry to quantify change was not available. Persistent field loss likely reflected residual structural injury of the occipital cortex/optic radiations despite resolution of inflammatory edema. Individualized immunosuppressive and prophylactic strategies, together with multidisciplinary care, were central to management.

Furthermore this case exemplifies the delicate therapeutic balance required in patients undergoing allo‑HSCT for acute leukemia. Intensive immunosuppression is essential to prevent or control graft‑versus‑host disease (GvHD), yet it delays T‑cell reconstitution and increases susceptibility to opportunistic infections. The development of cerebral toxoplasmosis, a rare but often fatal parasitic infection in non-HIV immunocompromised patients, illustrates this tension vividly. Despite profound CD4⁺ lymphopenia, our patient survived, highlighting the critical value of early imaging, pathogen-directed diagnostics, and aggressive therapy (including prolonged antimicrobial prophylaxis) in achieving a favorable outcome. His clinical course underscores that even severe CNS infections can be successfully managed with timely intervention and tailored care.

The incidence of visual field defects specifically attributable to toxoplasma encephalitis is not well quantified, as most reports focus on broader neurological presentations. However, focal deficits are common in toxoplasmic encephalitis and reflect lesion topography; involvement of the optic radiations or occipital cortex can produce isolated homonymous visual field loss. Case reports have documented homonymous hemianopic or quadrantopic defects as a presenting sign, supporting the need to include toxoplasmosis in the differential diagnosis of acute post-chiasmal visual field loss in immunocompromised patients [10, 11]. 

Ocular toxoplasmosis classically manifests as retinochoroiditis and may coexist with CNS disease, particularly in immunosuppressed hosts. In the present case, no posterior segment inflammation or retinochoroiditis was detected at presentation or on subsequent ophthalmic follow-up through May 2025. Later visual acuity decline was driven by chronic ocular GVHD–related ocular surface disease, underscoring the importance of differentiating neuro-ophthalmic from ocular surface contributors to vision loss [6, 9]. 

In this case, Pneumocystis prophylaxis with cotrimoxazole (TMP-SMX) was initially withheld due to concerns about hematologic toxicity, particularly thrombocytopenia. Instead, the patient received inhaled pentamidine, which offers protection against Pneumocystis jirovecii but does not protect against *Toxoplasma gondii*. In retrospect, this decision may warrant reevaluation, given the high mortality associated with cerebral toxoplasmosis in transplant recipients. Current guidelines emphasize individualized prophylaxis in high‑risk patients: all allo‑HSCT candidates should be screened for latent Toxoplasma infection, and seropositive patients should receive effective prophylaxis, typically with TMP‑SMX. For instance, transplant guidelines (e.g., CDC/IDSA recommendations) advise that all allogeneic HCT candidates be screened for latent T. gondii infection and that seropositive patients receive effective prophylaxis, typically with TMP-SMX. If TMP-SMX is contraindicated, anti‑toxoplasma prophylaxis (e.g., clindamycin plus pyrimethamine with leucovorin) or close monitoring (such as serial Toxoplasma PCR screening) should be implemented, as inhaled pentamidine alone provides no protection against toxoplasmosis [12, 13]. Furthermore, after treating active toxoplasmosis, continuing suppressive therapy for the duration of immunosuppression is recommended to prevent relapse [14]. Adhering to such evidence-based guidelines, while carefully balancing prophylactic benefits against potential toxicities, is crucial to mitigate the risk of life-threatening T. gondii reactivation as highlighted by this case.

## Conclusion

Homonymous visual field loss after allo-HSCT should prompt urgent neuroimaging and pathogen-directed diagnostics, as cerebral toxoplasmosis may present primarily with post-chiasmal neuro-ophthalmic deficits. Management requires a dynamic balance between maintaining adequate GvHD control and minimizing infection risk during delayed immune reconstitution, supported by multidisciplinary decision-making. In seropositive high-risk recipients, prophylaxis should ensure anti-toxoplasma coverage; when TMP-SMX is contraindicated, alternative strategies and/or close molecular surveillance should be implemented, and suppressive therapy should be continued during ongoing immunosuppression to reduce relapse risk.

### Learning points:

1. Cerebral toxoplasmosis is a rare but potentially fatal complication in non-HIV immunocompromised patients, particularly following allogeneic stem cell transplantation (allo-HSCT).
2. Long-term prophylaxis against opportunistic infections must be carefully balanced against hematologic toxicity; inhaled pentamidine does not protect against *Toxoplasma gondii*.
3. Persistent CD4⁺ lymphopenia may occur even months after cessation of systemic immunosuppression and warrants ongoing immune monitoring.
4. Neurological symptoms such as visual field loss or cognitive slowing in post-HSCT patients should prompt early MRI and CSF diagnostics to rule out opportunistic CNS infections.
5. Successful management of both chronic GVHD and severe infections requires multidisciplinary coordination and individualized immunosuppressive and prophylactic strategies.

## Data Availability

All data relevant to this report are included within the manuscript. Additional details are available from the corresponding author upon reasonable request.
